# Supplementary material for: Effects of Paper Mulberry Silage on the Milk Production, Apparent Digestibility, Antioxidant Capacity, and Fecal Bacteria Composition in Holstein Dairy Cows
Source: Animals (Basel). 2020 Jul 7;10(7):1152. doi: 10.3390/ani10071152 (PMC7401539; doi:10.3390/ani10071152)
Supplement: Supplementary file 1 [file animals-10-01152-s001.pdf]

Table S1. The chemical composition of paper mulberry silage

Item[1]	DM, as-fed basis	NDF	ADF	CP
Content,%	20.4	43.1	33.5	16.9

[1] DM: dry matter, NDF: neutral detergent fiber, ADF: acid detergent fiber, CP: crude protein.

Table S2. Effects of paper mulberry silage on apparent total tract digestibility of dairy cow.

**Item[1]**	**Period**	**Treatment[2]**			**SEM**	***p*-value**
		**CON**	**PM1**	**PM2**		
DM, %	I	70.9	70.1	69.3	2.7	0.97
	II	72.1	72.1	71.1	1.5	0.94
NDF, %	I	68.9	69.0	69.0	1.2	0.53
	II	61.4	59.5	57.8	1.1	0.19
ADF, %	I	53.5	50.4	51.3	1.1	0.77
	II	50.3	50.4	50.3	1.7	0.77
CP, %	I	73.6	74.4	72.6	1.5	0.39
	II	72.0	71.7	71.1	1.8	0.19

[1] DM: dry matter, NDF: neutral detergent fiber, ADF: acid detergent fiber, CP: crude protein.

[2] CON: the control group fed none paper mulberry silage diet; PM1 and PM2: the treatment groups fed 4.5 and 9.0% paper mulberry silage supplementary diet during the period I. Then, treatment groups fed 13.5 and 18.0% paper mulberry silage during the second period, respectively. Period I, the first 28 days; Period II, the second 28 days.

Table S3. Effects of paper mulberry silage on the feces microbial OUT, richness and diversity of cows

**Items**	**Period**	**Treatment**			**SEM**	***p*-values**
		**CON**	**PM1**	**PM2**		
OTU number	I	1979.8	2023.2	1958.8	15.7	0.60
	II	2111.0	2093.8	1981.2	33.1	0.35
Richness estimate						
Chao1	I	2477.7	2609.9	2638.8	40.4	0.12
	II	2678.3	2708.2	2582.7	30.9	0.52
Ace	I	2422.2	2555.4	2584.5	40.8	0.11
	II	2624.2	2651.6	2554.0	23.6	0.60
Diversity indices						
Shannon	I	8.1	8.2	7.8	0.1	0.07
	II	8.2	8.0	7.9	0.1	0.34
Simpson	I	1.0	1.0	1.0	<0.1	0.11
	II	1.0	1.0	1.0	<0.1	0.42

CON: the control group fed none paper mulberry silage diet; PM1 and PM2: the treatment groups fed 4.5 and 9.0% paper mulberry silage supplementary diet during the period I. Then, treatment groups fed 13.5 and 18.0% paper mulberry silage during the second period, respectively. Period I, the first 28 days; Period II, the second 28 days.

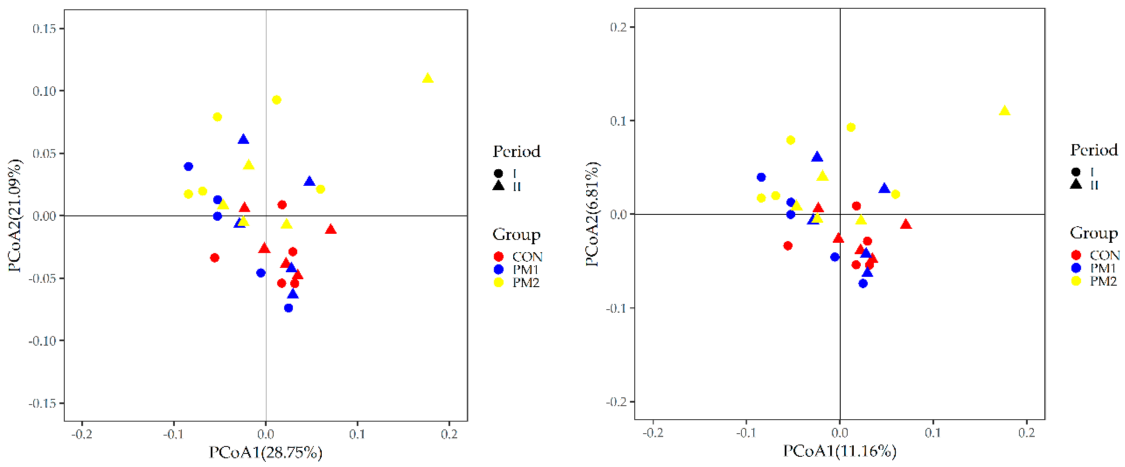

(a) (b)

Fig. S1. Weighted (a) and unweighted (b) principal coordinate analysis (PCoA) illustrating relationships among fecal bacterial populations in cows fed different diets

CON: the control group fed none paper mulberry silage diet; PM1 and PM2: the treatment groups fed 4.5 and 9.0% paper mulberry silage supplementary diet during the period I. Then, treatment groups fed 13.5 and 18.0% paper mulberry silage during the second period, respectively. Period I, the first 28 days; Period II, the second 28 days.

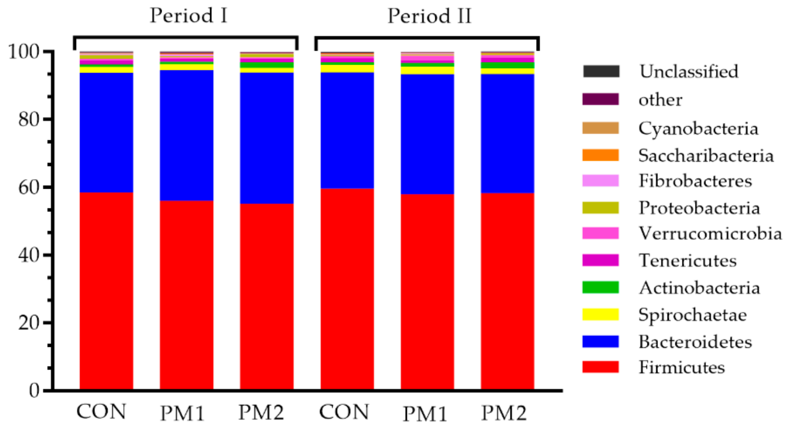

Fig. S2. Fecal bacteria composition at phylum level of dairy cows with different diets

CON: the control group fed none paper mulberry silage diet; PM1 and PM2: the treatment groups fed 4.5 and 9.0% paper mulberry silage supplementary diet during the period I. Then, treatment groups fed 13.5 and 18.0% paper mulberry silage during the second period, respectively. Period I, the first 28 days; Period II, the second 28 days.
